# Mitofusin2, as a Protective Target in the Liver, Controls the Balance of Apoptosis and Autophagy in Acute-on-Chronic Liver Failure

**DOI:** 10.3389/fphar.2019.00601

**Published:** 2019-05-31

**Authors:** Ran Xue, Jing Yang, Lin Jia, Xuemin Zhu, Jing Wu, Yueke Zhu, Qinghua Meng

**Affiliations:** Department of Critical Care Medicine of Liver Disease, Beijing You-An Hospital, Capital Medical University, Beijing, China

**Keywords:** mitofusin2, acute-on-chronic liver failure, autophagy, apoptosis, BNIP3

## Abstract

**Aim:** Acute-on-chronic liver failure (ACLF) is closely related to mitochondrial dysfunction. Previous studies showed the vital role of mitofusin2 (Mfn2) in the regulation of mitochondrial function. However, the effect of Mfn2 on ACLF remains unknown. As one of mitochondrial-related pathways, BNIP3-mediated pathway controls the balance between apoptosis and autophagy. However, the relationship between Mfn2 and BNIP3-mediated pathway in ACLF is still obscure. The aim of our study is to clarify the effect of Mfn2 and potential molecular mechanisms in ACLF.

**Methods:** We collected liver tissue from ACLF patients and constructed an ACLF animal model and a hepatocyte autophagy injury model, using adenovirus and lentivirus to deliver Mfn2 and Mfn2-siRNA to liver cells, in order to assess the effect of Mfn2 on autophagy and apoptosis in ACLF. We explored the biological mechanisms of Mfn2-induced autophagy and apoptosis of ACLF through Western blotting, Quantitative Real-Time PCR (RT-PCR), transmission electron microscopy, immunofluorescence, immunohistochemical staining, and hematoxylin–eosin staining.

**Results:** Compared with the normal liver tissue, the expressions of Mfn2, Atg5, Beclin1, and LC3-II/I were significantly decreased and the expression of P62 was much higher in patients with ACLF. Mfn2 significantly attenuated ACLF, characterized *via* microscopic histopathology and reduced serum AST and ALT levels. Mfn2 promoted the expressions of ATP synthase β, Atg5, Beclin1, LC3-II/I, and Bcl2 and reduced the expressions of P62, Bax, and BNIP3.

**Conclusions:** Mfn2 plays a protective role in the progression of ACLF. BNIP3-mediated signaling pathway is not the only factor associated with Mfn2 controlling the balance of apoptosis and autophagy in ACLF. Mfn2 will provide a promising therapeutic target for patients with ACLF.

## Introduction

Acute-on-chronic liver failure (ACLF) is an acute decompensation of chronic liver disease complicated with other organ failure (Olson, 2019). Despite progression of clinical treatments, ACLF is still associated with increased mortality and morbidity (Xue et al., 2017a; Xue et al., 2017b). It is one of the mainly causes for clinical problems that pathogenesis and progression of ACLF are not fully understood. Therefore, an in-depth exploration into the progression of ACLF is urgently needed to develop novel therapies.

The mitochondrion is one of the most important organelles for energy metabolism, and its dysfunction is closely related to the incidence of liver failure (Mansouri et al., 2018; Waltz et al., 2018). It performs its biological functions through continuous division and fusion (Xue et al., 2018), and many proteins take part in these processes, such as mitofusin 1 (Mfn1), optic atrophy 1 (OPA1), dynamin-related protein (DRP1), and mitochondrial fission 1 protein (Fis1) (Singh et al., 2016a; Singh et al., 2016b; Sacerdoti et al., 2018).

In 2001, Santel and Fuller (2001) found a gene in lung tissue, which was confirmed to promote mitochondrial fusion, and named mitochondrial fusion protein 2 (Mfn2). Mfn2 is located in the outer membrane of mitochondria and mainly promotes the outer membrane fusion and endomembrane docking (Kumar et al., 2016). Many studies have demonstrated the vital effects of Mfn2 on cell apoptosis and autophagy. Interestingly, Mfn2 plays distinct roles in apoptosis and autophagy among different tissues (Karbowski et al., 2002; Xue et al., 2018). Until now, the effect of Mfn2 on apoptosis and autophagy remains unknown in ACLF.

Both apoptosis and autophagy are well-controlled biological processes that are essential for disease, tissue homeostasis, and development. Interactions among components of these two pathways suggest a complex cross-talk, which are usually induced *via* similar stimuli (Baines, 2010; Booth et al., 2014). The mitochondrion is a key regulator of apoptosis. In response to stress, BH3-only proteins can activate pro-apoptotic proteins Bak and Bax. Mitochondrial autophagy targets dysfunctional mitochondria for degradation through lysosomes and undergoes extensive cross-talk with apoptosis signaling (Choe et al., 2015). BNIP3 integrates mitophagy and apoptosis signaling at different signaling domains. Meanwhile, BNIP3 suppresses pro-survival Bcl2 members by its BH3 domain and activates mitophagy *via* its LC3 interacting region (LIR). BNIP3-mediated mitophagy prior to apoptosis induction can inhibit mitochondrial activation of caspases, indicating that the reduction to mitochondrial levels may be a pro-survival factor (Baines, 2010; Zhu et al., 2013; Choe et al., 2015). Therefore, as one of mitochondrial-related pathways, BNIP3-mediated pathway has an effect on controlling the balance of apoptosis and autophagy. However, there are few studies related to the BNIP3-mediated pathway in ACLF at present. Meanwhile, the relationship between Mfn2 and BNIP3-mediated pathway in ACLF remains obscure.

In this study, we collected the liver tissue from ACLF patients and constructed a hepatocyte injury autophagy model and an ACLF animal model, using Mfn2-adenovirus (Ad-Mfn2), Mfn2-lentivirus (LV-Mfn2), and Mfn2-siRNA to interfere the expression of Mfn2, so as to clarify the relationship among Mfn2, apoptosis, autophagy, and BNIP3-mediated pathway in ACLF. It will provide the theoretical basis to understand the progression of ACLF and develop a new target for clinical therapy in patients with ACLF.

## Materials and Methods

The investigation was conducted according to the ethical standards and the Declaration of Helsinki. All methods and procedures related to animals were performed following the guidelines of the Animals Committee. The research was permitted by the Ethical Committee of Beijing You-An Hospital, Capital Medical University. All study patients gave their informed consent to participate in the study.

### Hepatocyte Autophagy Injury Model and Intervention Treatment

The human hepatocyte cell line L02 was grown in DMEM containing 10% fetal bovine serum (FBS), streptomycin (100 mg/ml), and penicillin (100 U/ml). The L02 cells treated with serum-free medium for 48 h were used to construct hepatocyte autophagy injury model. The cell experiments were divided into four groups. The control group was L02 cells treated with 10% FBS medium for 48 h. The model L02 group was L02 cells treated with serum-free medium for 48 h. In the intervention groups, the L02 cells were pre-incubated with Mfn2-adenovirus (Ad-Mfn2) or Mfn2 siRNA for 48 h and then treated with serum-free medium for 48 h. Lipofectamine 3000 transfection reagent (Thermo Fisher) was used for the transfection of siRNA. Small interfering RNAs (siRNA) specific to Mfn2 and adenovirus encoding the Mfn2 open reading frame (Ad-Mfn2), as well as nonspecific controls were constructed by JI KAI Gene Technology Co. Ltd. (Beijing, China).

### Animal Models of ACLF and Intervention Treatment

Healthy male SD rats (160–180 g) fed in the medical research center of Beijing You-an Hospital, Capital Medical University were used in our experiments. They were acclimated for 2 weeks before experimentation. The ACLF animal model was established according to a published method (Chen et al., 2011). Lentivirus encoding the Mfn2 (LV-Mfn2) and control lentivirus were constructed by JI KAI Gene Technology Co. Ltd. (Beijing, China). Animals were randomly divided into six groups (N = 10). The grouping and treatment regimen were as follows: Group-I, normal control [the rats received normal saline (1.5 ml/kg), twice per week, 10 weeks)]; Group-II, ACLF model [the rats intraperitoneally received vegetable oil and 40% CCL4 mixture (1.5 ml/kg), twice per week, 10 weeks]; Group-III, LV-Mfn2 group [the rats received vegetable oil and 40% CCL4 mixture (1.5 ml/kg), twice per week and LV-Mfn2 (1 × 10^9^ TU/ml), 200  μl per one rat, once per 3 weeks, 10 weeks]; Group-IV, LV-Con group [the rats received vegetable oil and 40% CCL4 mixture (1.5 ml/ kg), twice per week, and LV-Con (1 × 10^9^ TU/ml), 200 μl per one rat, once per 3 weeks, 10 weeks]; Group-V, siRNA group [the rats received vegetable oil and 40% CCL4 mixture (1.5 ml/kg), twice per week, and siRNA (1 × 10^9^ TU/ ml), 200 μl per one rat, once per 3 weeks, beginning from the sixth week]; Group-VI, siRNA-NC group [the rats received vegetable oil and 40% CCL4 mixture (1.5 ml/kg), twice per week, and siRNA-NC (1 × 10^9^ TU/ml), 200 μl per one rat, once per 3 weeks, beginning from the sixth week]; and Group II-Group VI [on the basis of chronic liver injury, the rats were then challenged intraperitoneally with Lipopolysaccharides (LPS) (100 μg/kg) combined with D-gal (0.5 g/kg) to induce acute liver failure].

### Patient Selection

A total of five patients who were diagnosed as ACLF and underwent liver transplantation from February 2016 to January 2018 were included in this study. Normal liver tissues were obtained from liver transplant donors as the normal control group.

The entry criteria included the following: all enrolled patients met the criteria of ACLF from the consensus recommendations of the Asian Pacific Association for the Study of the Liver (APASL) (Sarin et al., 2014).

**Figure 1:**
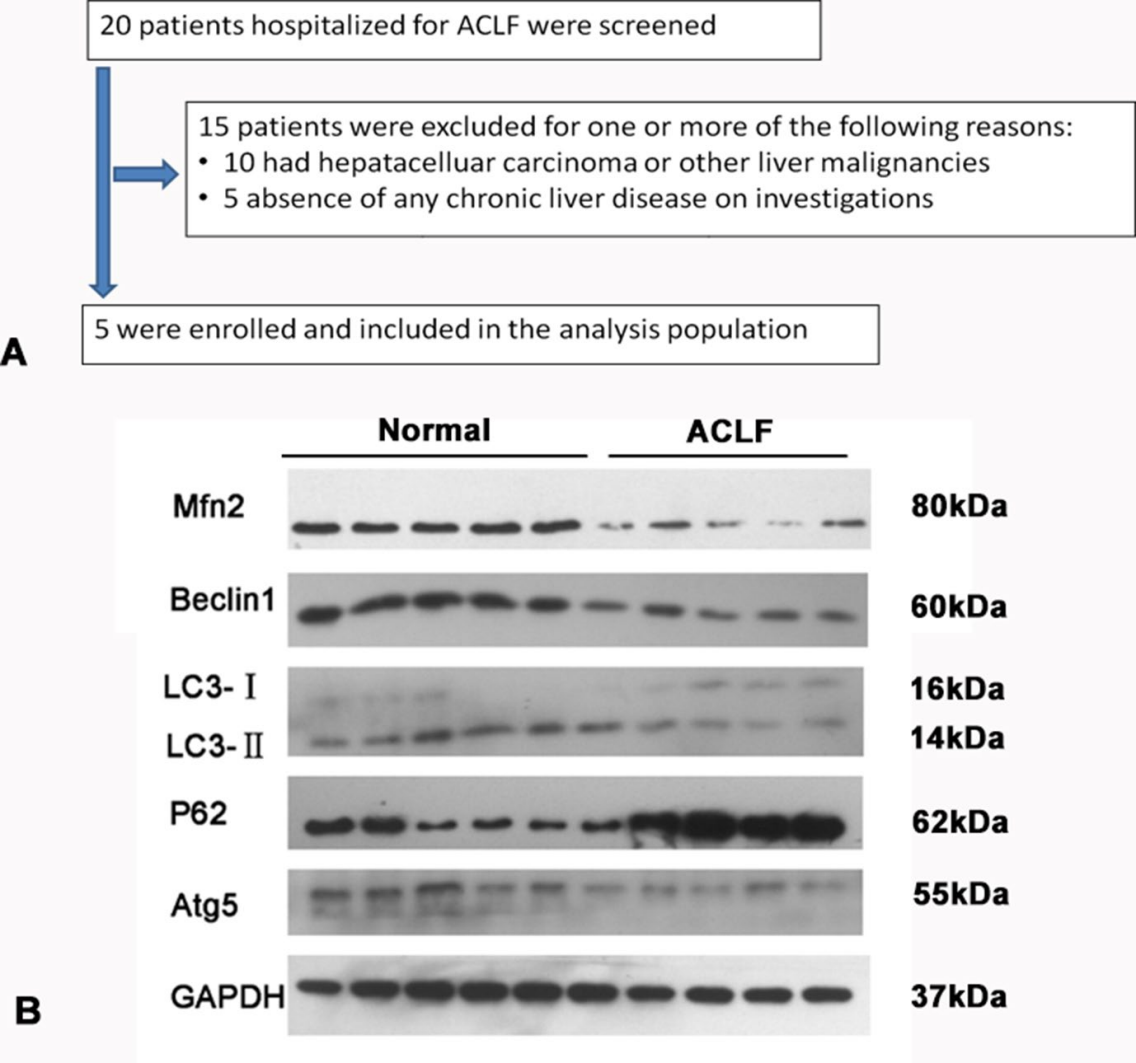
**(A)** Study flow: diagram showing the process of study selection and exclusion in of ACLF patients. **(B)** Western blotting analysis for Mfn2, LC3-II/I, p62/SQSTM1, Beclin1, and Atg5 expressions in the ACLF patients and normal liver tissue.

The exclusion criteria were the following: combined with liver cancer, known decompensated cirrhosis prior to onset of acute hepatic insult, jaundice induced *via* hemolytic jaundice and obstructive jaundice, age less than 18 years, absence of any chronic liver disease on investigations, and prolonged prothrombin time induced by blood system diseases were also excluded. The flowchart for the selection of ACLF patients is shown in **Figure 1A**.

### Immunofluorescence

Immunofluorescence analysis was performed following a previous study (Cummins et al., 2018). P62 immunoreactivity was detected with a fluorescence microscope (Nikon Eclipse 80i, Tokyo, Japan). More than 10^3^ cells were counted to quantitate cell autophagy.

### Quantitative Real-Time PCR

Guided by the manufacturer’s instructions, RNeasy Mini Kit (QIAGEN, Germany) was used to isolate total RNA and PrimeScript RT reagent kit (Takara Bio Inc, Japan) was used to perform RT-PCR assay (Xue et al., 2017a; Xue et al., 2017b). The primer pairs used to detect GAPDH, Mfn2, Beclin1, Atg5, Bax, Bcl2, LC3-I, and LC3-II were synthesized by Shanghai Sangon biotech (Shanghai, China) (**Supplementary Table S1** online).

### Western Blot Analysis

Western blot analysis was performed as depicted in a previous study (Serna-Salas et al., 2018). The primary antibodies (GAPDH, Mfn2, and P62 from Abcam; Beclin1, Atg5, and BNIP3 from Cell Signaling Technology; ATP synthase β and LC3 from Sigma) are listed in **Supplementary Table S2** online. X-ray film was used to record the protein bands.

### Hematoxylin–Eosin Staining

Livers were fixed in 10% buffered formalin, dehydrated with graded ethanol, and embedded in paraffin. Five-micrometer paraffin sections were mounted on glass slides and stained by hematoxylin–eosin (H&E) for light microscopy examination. The details of routine H&E staining followed a previous study (Xue et al., 2017a; Xue et al., 2017b).

### Immunohistochemical Staining and Assessment

Immunohistochemical (IHC) staining was performed as depicted in a previous research (Xue et al., 2017a; Xue et al., 2017b). The primary antibodies are shown in **Supplementary Table S2** online. Sections were incubated *via* horseradish peroxidase-conjugated antibody. Slides were counterstained in hematoxylin and photographed using a digital camera-aided computer system (Nikon digital camera, Japan). The expression of protein was assessed independently and blindly through two investigators. According to the number of positive cells to analyze the expression of protein, the IHC results were categorized as follows: +, positive (> 30%); ±, weakly positive (10–30%); −, negative (< 10%).

### Liver Function Test

Serum levels of alanine aminotransferase (ALT) and aspartate aminotransferase (AST) were measured with an AU400 automatic biochemical analyzer.

### Transmission Electron Microscopy

Liver tissues were fixed and embedded in Epon-Araldite resin. Next, ultrathin sections were stained with uranyl acetate and 0.3% lead citrate and examined under a transmission electron microscope at 80 kV (JEM-1200; Jeol Ltd., Tokyo, Japan).

### Caspase-3 Activity Measurement

The activity of caspase-3 was judged with the Caspase-3 Activity Assay Kit (C1115, Beyotime, China). The absorbance (A405) was evaluated with an ELISA reader (BioTeck, USA) and converted to the amounts of pNA.

### Reactive Oxygen Species Measurement

Changes in intracellular reactive oxygen species (ROS) levels were detected through the oxidative conversion of cell-permeable 2’,7’-dichlorofluorescein diacetate (DCFH-DA) to fluorescent di-chlorofluorescein (DCF). DCF fluorescence was tested with a FACScan flow cytometer (BectonDickinson).

### Statistical Analyses

All data represent at least three independent experiments. Statistical significance was calculated by Student’s t-test (SPSS19.0, SPSS Inc., Chicago, IL, USA). Quantitative variables are shown as mean ± S.D. P < 0.05(*) was considered statistically significant.

## Results

### The Expression of Mfn2 in the ACLF Patients

Compared with the normal liver tissue, the expression of Mfn2 was significantly decreased in ACLF patients. It seems that Mfn2 may play a role during the development of ACLF. As for the level of autophagy, the expressions of Atg5, Beclin1, and LC3-II/I were significantly lower and the expression of P62 was much higher in ACLF patients (**Figure 1B**). It suggested that cell autophagy was inhibited in patients with ACLF.

### Mfn2 Promoted the Level of Autophagy in ACLF

**Figure 2:**
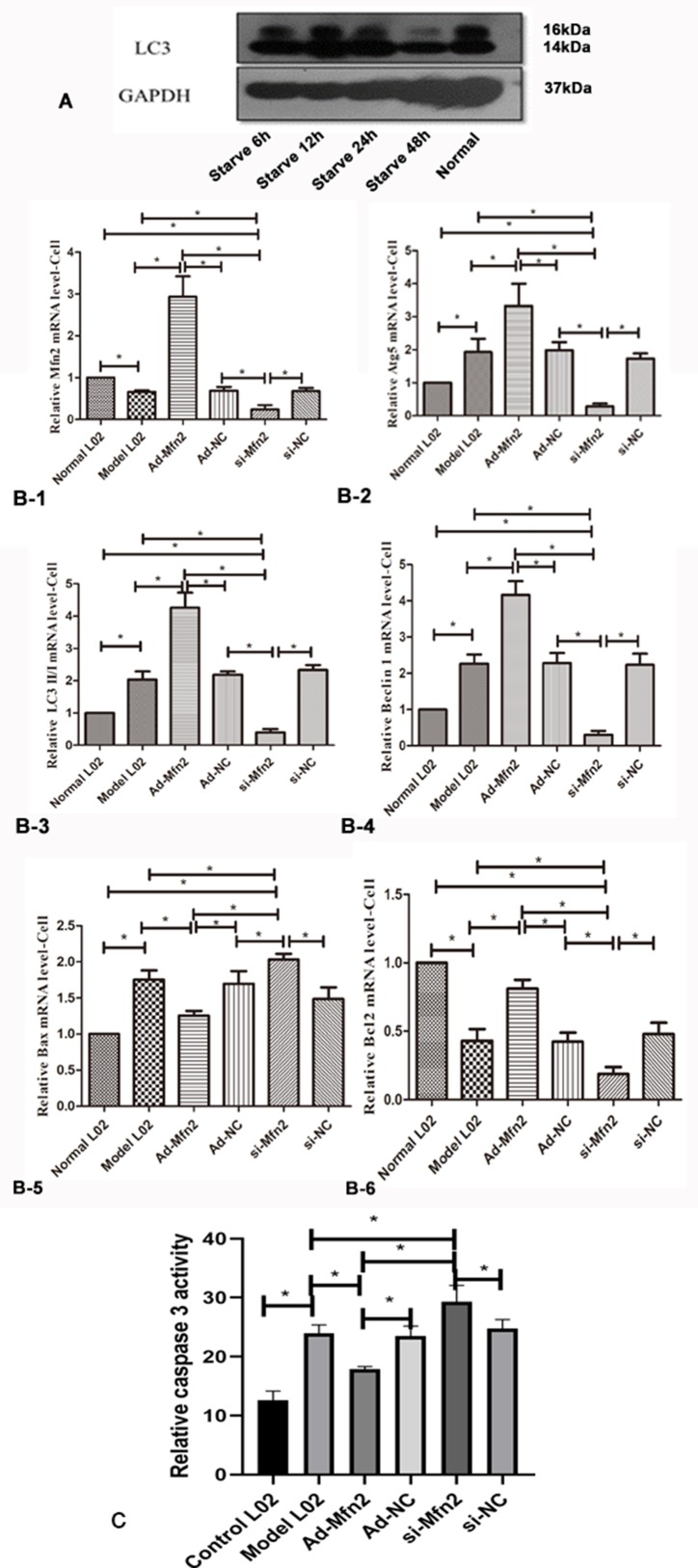
**(A)** Western blotting analysis for LC3-II/I expression of L02 cells was performed after 6, 12, 24, and 48 h with serum-free medium. **(B-1)** Real-time RT-PCR analysis for Mfn2; **(B-2)** Real-time RT-PCR analysis for Atg5; **(B-3)** Real-time RT-PCR analysis for LC3II/I; **(B-4)** Real-time RT-PCR analysis for Beclin1; **(B-5)** Real-time RT-PCR analysis for Bax; **(B-6)** Real-time RT-PCR analysis for Bcl2 (*P < 0.05, N = 3). **(C)** The decreased caspase-3 activity was observed in Ad-Mfn2 groups compared with the ACLF model group (*P < 0.05, N = 4).

In order to explore the effect of Mfn2 on autophagy in liver cells, the starvation treatment of L02 cells with serum-free medium was used to construct a hepatocyte autophagy injury model. L02 cells had a great effect on cell autophagy after 48 h with serum-free medium; we proceeded follow-up researches in L02 cells after 48 h with serum-free medium (**Figure 2A**).

**Figure 3:**
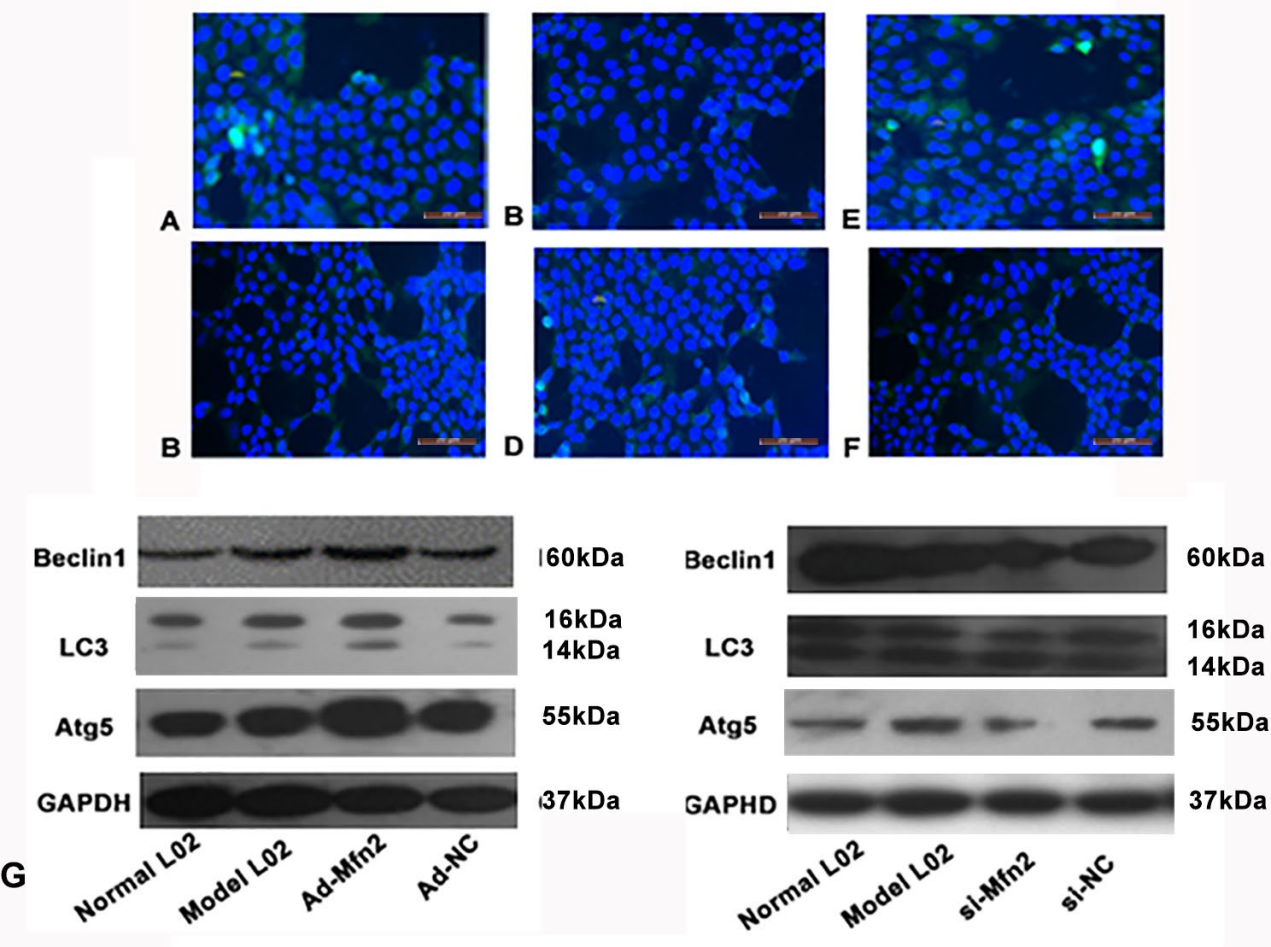
The expression of P62 was tested using fluorescence microscopy in different groups. **(A)** Normal L02 group. **(B)** Model L02 group. **(C)** Ad-Mfn2 group. **(D)** Ad-Mfn2 negative control group. **(E)** si-Mfn2 group. **(F)** si-Mfn2 negative control group. Original magnification, ×200. **(G)** Western blotting analysis for Beclin1, Atg5, and LC3-II/I expressions of L02 cells was performed after 48 h with serum-free medium in different groups.

The RT-PCR results showed that in the Ad-Mfn2 group, the expressions were significantly increased for Atg5, Beclin1, and LC3-II/I mRNA levels compared with the Model-L02 and Mfn2-siRNA groups. Meanwhile, in the Mfn2-siRNA group, the expressions of Atg5, Beclin1, and LC3-II/I mRNA levels were much lower than those in the Model-L02 group (**Figure 2B**). Western blotting performed the same results in the hepatocyte autophagy injury model (**Figure 3G**).

Immunofluorescence was used to observe the changes of autophagy *via* P62 levels in the hepatocyte autophagy injury model. The green fluorescent spots of P62 in the Ad-Mfn2 group significantly decreased compared with other groups. Meanwhile, compared to other groups, further increased level of green fluorescent spots of P62 can be observed in the Mfn2-siRNA group (**Figure 3A–F**).

**Figure 4:**
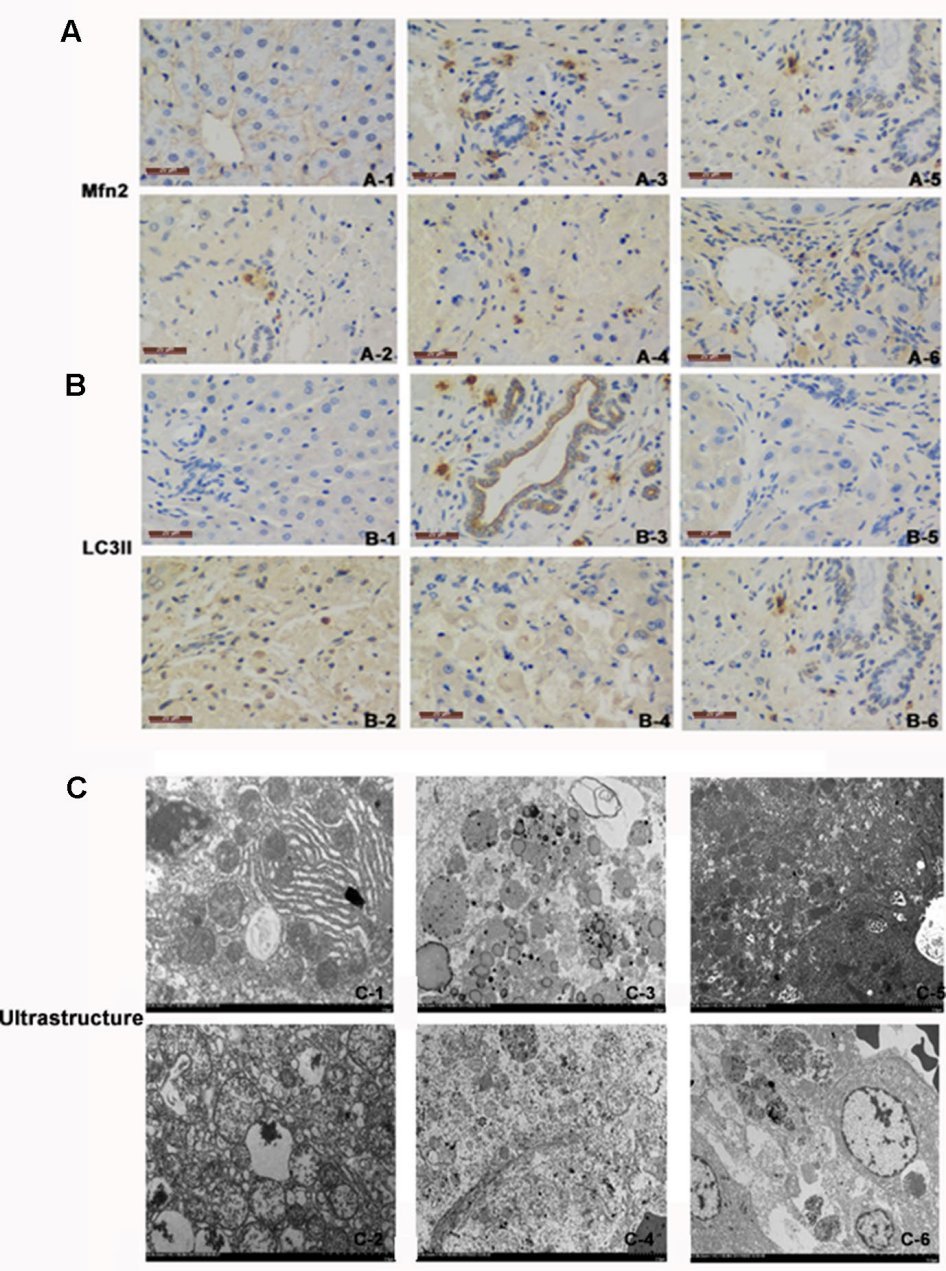
**(A)** There was immunostaining for Mfn2 in different groups. Mfn2 was stained brown. **(A-1)** Normal group. **(A-2)** ACLF animal model group. **(A-3)** LV-Mfn2 group. **(A-4)** LV-Mfn2 negative control group. **(A-5)** si-Mfn2 group. **(A-6)** si-Mfn2 negative control group. Original magnification, ×400. **(B)** There was immunostaining for LC3II in different groups. LC3II was stained brown. **(B-1)** Normal group. **(B-2)** ACLF animal model group. **(B-3)** LV-Mfn2 group. **(B-4)** LV-Mfn2 negative control group. **(B-5)** si-Mfn2 group. **(B-6)** si-Mfn2 negative control group. Original magnification, ×400. **(C)** Ultrastructure of the liver *via* electron microscopy. **(C-1)** Normal group. **(C-2)** ACLF animal model group. **(C-3)** LV-Mfn2 group. **(C-4)** LV-Mfn2 negative control group. **(C-5)** si-Mfn2 group. **(C-6)** si-Mfn2 negative control group. Original magnification, ×5,000.

Electron microscopy is the gold standard for ultrastructure. Under the electron microscope, the mitochondrial mites were clear and the mitochondrial membrane was intact in the normal control group. In the ACLF animal group and the LV-Mfn2-siRNA group, most of the mitochondrial contents had been degraded, and the mitochondria were vacuolated with endoplasmic reticulum expansion. The number of autophagosomes or autophagic lysosomes in the LV-Mfn2 group was significantly higher than in the LV-Mfn2-siRNA group and the ACLF animal group. Meanwhile, most of the mitochondrial structure remained and the mitochondrial membrane integrity was poor with increased electron density in the LV-Mfn2 group (**Figure 4**).

In IHC staining, Mfn2 was located on the outer membrane of mitochondria. In the normal control group, only a small amount of Mfn2 was expressed in the cytoplasm of hepatic parenchyma cells. Compared with the ACLF animal group and the LV-Mfn2 negative control group, a large amount of Mfn2 was expressed in the cytoplasm of hepatic parenchyma cells in the LV-Mfn2 group, which indicated that Mfn2 was successfully overexpressed in rats. The expression of Mfn2 decreased significantly in the si-Mfn2 group, suggesting that Mfn2 was successfully knocked down in rats. LC3II mainly distributed in the cytoplasm of hepatocytes. In the normal control group, LC3II was lightly colored and less expressed. In the ACLF animal group, LC3II was darker and highly expressed than in the normal control group, which indicated that the level of autophagy increased when liver was damaged. The expression of LC3II in the LV-Mfn2 group was higher than in the LV-Mfn2-NC group. The expression of LC3II in the si-Mfn2 group was significantly less expressed than in the other groups. These results suggested that Mfn2 can promote autophagy in ACLF (**Figure 4**).

### Mfn2 Improved the Liver Tissue Status of ACLF

**Figure 5:**
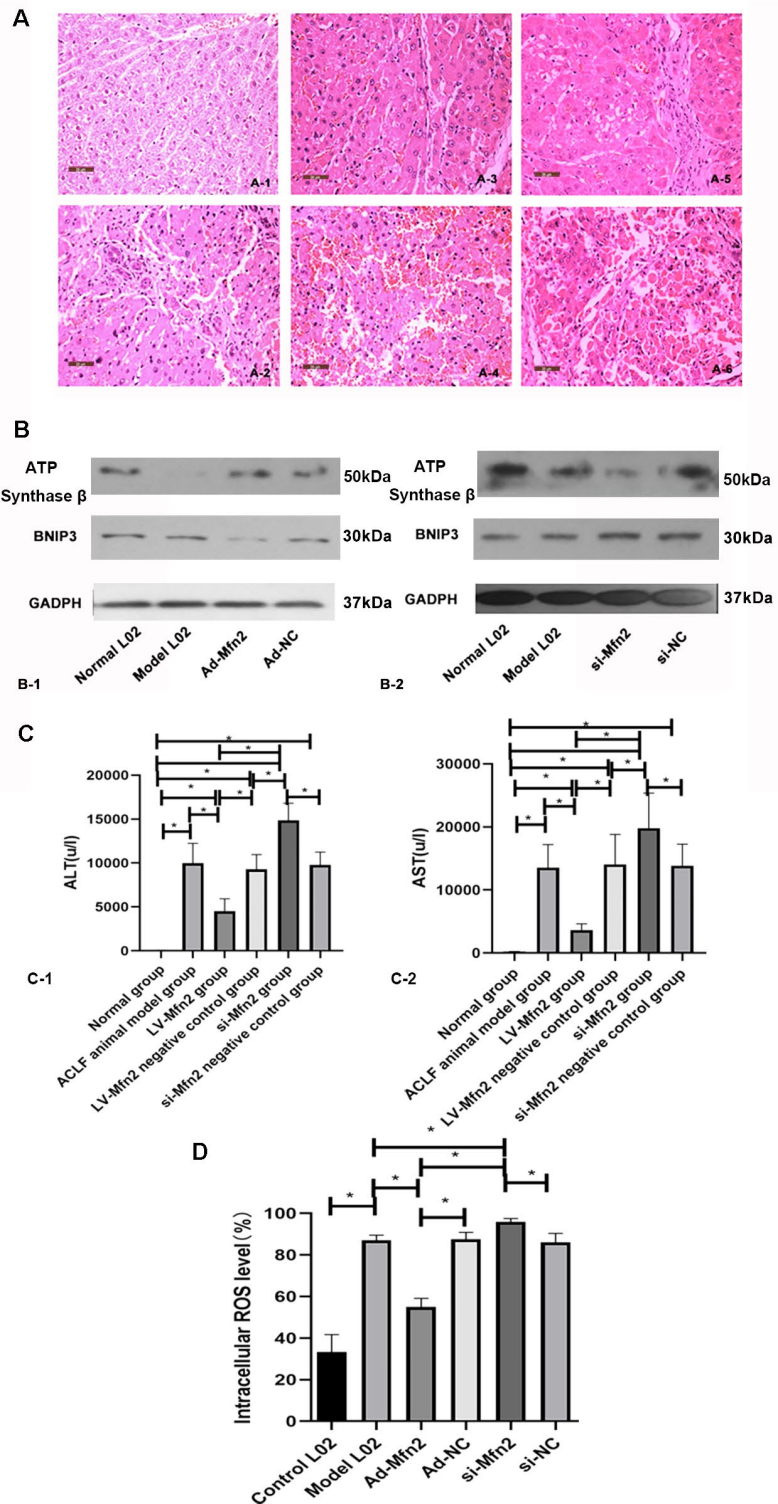
**(A)** There were typical H&E-stained sections in different groups. **(A-1)** Normal group. **(A-2)** ACLF animal model group. **(A-3)** LV-Mfn2 group. **(A-4)** LV-Mfn2 negative control group. **(A-5)** si-Mfn2 group. **(A-6)** si-Mfn2 negative control group. Original magnification, ×400. **(B)** Western blotting analysis for BNIP3 and ATP synthase β expressions of L02 cells was performed after 48 h with serum-free medium in different groups. **(C)** The effect of Mfn2 on liver function in ACLF. **(C-1)** ALT. **(C-2)** AST. **(D)** The effect of Mfn2 on intracellular ROS level.

H&E staining was used to evaluate the liver tissue status in the ACLF animal. In the normal control group, the structure of hepatic lobules was clear and intact. The hepatic plates and sinuses were arranged radially. The hepatocytes were polygonal. No inflammatory cells infiltrated. The structures of interlobular veins and interlobular bile ducts in the portal area were visible. Massive hepatocyte necrosis and sinusoidal expansion with congestion, occasional residual hepatocytes, and hepatocyte eosinophilia with a large number of neutrophil infiltration were observed in the ACLF animal group and the LV-Mfn2-siRNA group. The necrotic area of liver was nearly 100%, and most of the hepatic lobules disappeared. In the LV-Mfn2 group, hepatocyte necrosis was still seen, but the necrotic area was about 80%. Fiber septum could be seen, and most hepatic lobules were intact (**Figure 5A**). These results suggested that Mfn2 alleviated the histopathological changes of ACLF.

The serum AST and ALT are markers for liver function. Compared with the normal control group, the levels of ALT and AST significantly increased in the other groups, indicating that the liver function of each treatment group was damaged. The levels of ALT and AST in the LV-Mfn2 group were lower than in the ACLF group and the LV-Mfn2 negative control group (P < 0.05), suggesting that Mfn2 overexpression attenuated the degree of liver injury in ACLF (**Figure 5C**).

### Mfn2 Reduced Apoptosis in the Hepatocyte Autophagy Injury Model

Bcl2 and Bax were used to prove the level of cell apoptosis *via* RT-PCR. The expression of Bax mRNA was much lower in the Ad-Mfn2 group than in the ACLF model group and Mfn2-siRNA group. In the Ad-Mfn2 group, the expression of Bcl2 mRNA was significantly increased compared with the Mfn2-siRNA group and model group (**Figure 2B**). The caspase-3 activity was also used to evaluate the level of cell apoptosis. The decreased caspase-3 activity was discovered in the Ad-Mfn2 group compared with the ACLF model group (P < 0.05) (**Figure 2C**). All data indicated that Mfn2 inhibited cell apoptosis in the hepatocyte autophagy injury model.

### The Effects of Mfn2 on ATP Formation and ROS Generation

Compared with normal group, the expression of ATP synthase β was lower in the ACLF model. In the Ad-Mfn2 group, there was a higher expression of ATP synthase beta compared with the ACLF model. Meanwhile, si-Mfn2 decreased the expression of ATP synthase β compared with the ACLF group (**Figure 5B**). There was a significant decrease of ROS-positive cells in the Ad-Mfn2 group compared to the ACLF model. In the si-Mfn2 group, the average rate of DCF-positive cells was much higher than in the ACLF model (P < 0.05) (**Figure 5D**). It indicated that reduced Mfn2 expression in liver tissue led to an increase in ROS generation and a decrease in ATP synthesis, thereby increasing oxidative stress and disrupting metabolism in ACLF.

### The Relationship Between Mfn2 and BNIP3 in the Hepatocyte Autophagy Injury Model

In the ACLF model group, the expression of BNIP3 was increased compared with the normal group. BNIP3 was lower in the Ad-Mfn2 group and higher in the Mfn2-siRNA group compared to the ACLF model group. It demonstrated that Mfn2 reduced the expression of BNIP3 in the hepatocyte autophagy injury model (**Figure 5B**). As a pro-apoptotic protein, BNIP3 also reflects the level of cell apoptosis. The expression of BNIP3 indicated that Mfn2 inhibited cell apoptosis in the hepatocyte autophagy injury model, which was consistent with the expression of Bax and Bcl2 mRNA. It also means that the BNIP3-mediated signaling pathway is a factor associated with Mfn2, controlling the balance of apoptosis and autophagy in ACLF.

Many studies have shown that BNIP3 can trigger mitochondrial depolarization, and mitochondrial depolarization is sufficient to cause autophagic death. However, in our study, Mfn2 promoted the level of autophagy in ACLF, which is opposite to the expression of BNIP3. It indicated that the BNIP3-mediated signaling pathway is not the only factor associated with Mfn2 controlling the balance of apoptosis and autophagy in ACLF. There may exist another signaling pathway that also helped Mfn2 to influence cell autophagy in the hepatocyte injury autophagy model.

## Discussion

ACLF causes significant mortality and morbidity in patients with cirrhosis and/or chronic liver disease (Xue et al., 2017a; Xue et al., 2017b). Mitochondrial damage plays an important role in the progression of ACLF (Baines, 2010). Further exploring the progression and seeking new effective targets are key to improve the successful rate of ACLF treatment. In this study, we first proposed that Mfn2 can control the balance of apoptosis and autophagy in ACLF, which plays a protective role in the progression of ACLF. Mfn2 was considered to perform anti-apoptotic and pro-autophagic functions in ACLF. BNIP3-mediated signaling pathway is a factor, but not the sole one, which was associated with Mfn2 controlling the balance of apoptosis and autophagy in ACLF.

Chien et al. (2011) found that during sepsis autophagy occurred transiently in hepatocytes at early stage, and the decline of autophagy at end stage may contribute to the liver failure. This result is consistent with our observation that the level of autophagy in patients with ACLF was significantly reduced. ACLF is the end-stage liver disease, which strives to activate cell autophagy. However, due to the depletion of autophagy substrate, autophagy is insufficiently characterized by abnormal mitochondria and damaged organelle accumulation (Xie et al., 2013). It also indicates that cell autophagy is helpful to maintain normal cellular function and prevent the progression of liver diseases.

In the liver, chronic diseases are accompanied by a large number of hepatocyte apoptosis, which is one of the important pathological characteristics in various types of liver injury (Wang, 2015). The intervention of hepatocyte apoptosis can delay the progression of diseases and reduce the incidence of liver failure. Autophagy is related to the degradation of cytoplasm and mitochondria, which affects mitochondrial circulation and regulates hepatocyte apoptosis through mitochondrial pathway (Rabinowitz and White, 2010; Wu et al., 2010). Many studies have confirmed that there is a dynamic balance between autophagy and apoptosis in the pathogenesis of diseases (Chen et al., 2010; Nikoletopoulou et al., 2013).

There are common signaling pathways and regulatory proteins between apoptosis and autophagy. BNIP3 plays a dual regulatory role in apoptosis and autophagy (Choe et al., 2015; Zhu et al., 2013). BNIP3 is a pro-apoptotic protein that belongs to the Bcl2 family and contains a single Bcl2 homology domain. BNIP3 dissociates Beclin-1 from the Beclin-1/Bcl2 complex by competing with Beclin-1 for binding with Bcl2. When excess BNIP3 is present to bind Bcl2, more Beclin-1 is free to induce cell autophagy (Maiuri et al., 2007). Our study was first involved in the expression of BNIP3 in ACLF. The expression of BNIP3 was higher in the ACLF group, which indicated that the BNIP3-mediated signaling pathway is correlated to the progression of ACLF. Our results also showed that Mfn2 enhanced autophagy and reduced apoptosis in ACLF. Meanwhile, Mfn2 decreased the expression of BNIP3. It is known that BNIP3 promotes autophagy and apoptosis (Baines, 2010). Therefore, our results demonstrated that BNIP3-mediated signaling pathway is a factor, but not the sole one, which is associated with Mfn2 controlling the balance of apoptosis and autophagy in ACLF.

As for other potential signaling pathways that are associated with Mfn2 controlling the balance of apoptosis and autophagy in ACLF, β-catenin signaling pathway can negatively regulate autophagy. During starvation stress, β-catenin is directly interacting with LC3 and hence is specifically targeted for degradation by autophagy. AKT signaling pathway mediates the inhibition of autophagy through a variety of ways. PTEN, the phosphatase and tensin homolog, decreases the level of PIP3 and initiates the formation of autophagosome.

This study also has some limitations. Further studies concerning knockdown experiments of BNIP3 in ACLF are required in the future. Meanwhile, another signaling pathway that helps Mfn2 to influence the cell autophagy is still needed to be found.

## Data Availability Statement

The datasets were generated for this study. The raw data supporting the conclusions of this manuscript will be made available by the authors, without undue reservation, to any qualified researcher.

## Ethics Statement

The animal study protocol and the human study have been conducted in accordance with the ethical standards and according to the Declaration of Helsinki and have been approved by Beijing You-an Hospital, Capital Medical University. All study participants gave their informed and written consent to participate in the study.

## Author Contributions

RX and LJ wrote the manuscript. QM conceived the idea for this study. YZ analyzed the data. XZ, JW and JY did the experiments. RX, LJ, and JY contributed equally to this work.

## Funding

The study was supported by the National Natural Science Foundation of China (No. 81470877) and Natural Science Foundation of Beijing Municipality (No.7192085).

## Conflict of Interest Statement

The authors declare that the research was conducted in the absence of any commercial or financial relationships that could be construed as a potential conflict of interest.
