# Supplementary material for: Mitofusin2, as a Protective Target in the Liver, Controls the Balance of Apoptosis and Autophagy in Acute-on-Chronic Liver Failure
Source: Front Pharmacol. 2019 May 31;10:601. doi: 10.3389/fphar.2019.00601 (PMC6561379; doi:10.3389/fphar.2019.00601)
Supplement: Supplementary file 1 [file Table_1.docx]

Supplementary Table S1 online

8 sets of primers were designed using the Primer Explorer version 4 software (Eiken Chemical Co., Ltd., Tokyo, Japan; http://primerexplorer.jp/elamp4.0.0/index.html) and synthesized by Shanghai Sangon Co., Ltd.

Sequences of the primers used in this study

| Primer | 5′-3′ | 5′-3′ | |
| --- | --- | --- | --- |
| Mfn2-RT | TTGGCTTTGCTCTGAAGTGA | CTGGGACATTTGCTCATCTG | |
| Beclin 1-RT | AGCCTCTGAAACTGGACACG | CCTCTTCCTCCTGGCTCTCT | |
| LC3 I-RT | GCCTGTCCTGGATAAGACCA | CCGTCTTCATCCTTCTCCTG | |
| LC3 II-RT | CCGAGAAGACCTTCAAGCAG | CCATTCACCAGGAGGAAGAA | |
| Atg5-RT | CTCTGCCTTGGAACATCACA | AGCGTCAGCTTCCTTCACAC | |
| GAPDH-RT | GGGTGTGAACCACGAGAAAT | ACTGTGGTCATGAGCCCTTC | |
| Bax-RT | GAGGCAACCTGACCAGAAAC | | ATGATGGCAGTGGAGGAAAG |
| Bcl2-RT | CTTGATTGAGCGAGCCTTTC | | TGGACTGCCCCAGAAAAATA |
