# Supplementary material for: Mitofusin2, as a Protective Target in the Liver, Controls the Balance of Apoptosis and Autophagy in Acute-on-Chronic Liver Failure
Source: Front Pharmacol. 2019 May 31;10:601. doi: 10.3389/fphar.2019.00601 (PMC6561379; doi:10.3389/fphar.2019.00601)
Supplement: Supplementary file 2 [file Table_2.docx]

Supplementary Table S2 online

Antibodies and conditions used for western blotting analyses.

| Antibody | Number | Species | Dilution | Source |
| --- | --- | --- | --- | --- |
| Atg5 | #12994 | Rabbit monoclonal antibody | 1:1000 | Cell Signaling Technology |
| LC3B | L7543 | Rabbit polyclonal antibody | 1:1000 | Sigma |
| GAPDH | Ab22555 | Rabbit polyclonal antibody | 1:1000 | Abcam |
| Beclin1 | #3495 | Rabbit monoclonal antibody | 1:1000 | Cell Signaling Technology |
| Atg5 | #12994 | Rabbit monoclonal antibody | 1:1000 | Cell Signaling Technology |
| Mitofusin-2 | ab124773 | Rabbit monoclonal antibody | 1:1000 | Abcam |
| ATP  Synthase β | A9728 | Mouse monoclonal antibody | 1:1000 | Sigma |
| P62 | ab109012 | Rabbit monoclonal antibody | 1:1000 | Abcam |
| BNIP3 | ab10433 | Mouse monoclonal antibody | 1:1000 | Abcam |
